# Viscoelastic Behavior and Phase Structure of High-Content SBS-Modified Asphalt

**DOI:** 10.3390/polym14122476

**Published:** 2022-06-17

**Authors:** Dongdong Yuan, Chengwei Xing, Wei Jiang, Jingjing Xiao, Wangjie Wu, Pengfei Li, Yupeng Li

**Affiliations:** 1 Key Laboratory for Special Area Highway Engineering of Ministry of Education, Chang’an University, Xi’an 710064, China; ddy@chd.edu.cn (D.Y.); xingcw@chd.edu.cn (C.X.); wwj@chd.edu.cn (W.W.); 2020021080@chd.edu.cn (P.L.); liyupeng@chd.edu.cn (Y.L.); 2 School of Highway, Chang’an University, South 2nd Ring Road Middle Section, Xi’an 710064, China; 3 School of Civil Engineering, Chang’an University, Xi’an 710064, China; xiaojj029@sina.com

**Keywords:** high-content polymer modified asphalt, SBS, viscoelastic behavior, phase structure

## Abstract

To investigate the effect of styrene-butadiene-styrene (SBS) modifier content on the viscoelastic behavior of SBS-modified asphalt (SBSMA) at different temperatures and phase structures, the star SBS modifier was chosen to fabricate seven types of SBSMA with different contents. Multiple stress creep recovery (MSCR), linear amplitude sweep (LAS), and low-temperature frequency sweep tests were adopted to study the influence of SBS modifier content on the viscoelastic performance of SBSMA at high to low temperatures. The SBSMA’s microstructure with different contents was investigated using a fluorescence microscope. The results indicated that the change in non-recoverable creep compliance and creep recovery rate was bounded by 4.5% content at high temperatures, with an apparent turning point. The changing slope of content at less than 4.5% was much higher than that of the content greater than 4.5%. At medium temperatures, the fatigue life of SBSMA increased exponentially with the rising modifier content. The rate of increase in fatigue life was the largest as the content increased from 4.5% to 6.0%. At low temperatures, the low-temperature viscoelastic property index *G* (60 s) of SBSMA decreased logarithmically as the modifier content increased. In terms of the microscopic phase structure, the SBS modifier gradually changed from the dispersed to the continuous phase state with the increasing SBS modifier content.

## 1. Introduction

**Figure 1:**
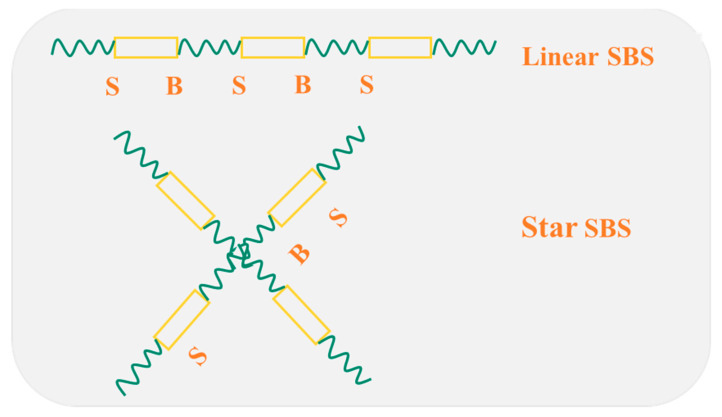
Linear SBS and Star SBS.

Asphalt pavement consists of aggregates, fillers, and asphalt binders; the design of asphalt binders and research on its related properties remain at the core of asphalt pavement [1,2]. Using an asphalt binder with excellent properties can remarkably improve the quality of asphalt pavement [3,4]. With the increasing traffic load, a neat asphalt binder will not be enough to fulfill the requirements of traffic development [5,6]. Styrene-butadiene-styrene (SBS)–modified asphalt (SBSMA) is universally applied because of its properties such as excellent durability, anti-ageing, fatigue resistance, and water damage resistance [7,8]. SBSMA has been utilized extensively on national highways in China [9,10]. SBS is a block copolymer created from the anionic polymerization of 1,3-butadiene, styrene (monomer), tetrahydrofuran (activator), and n-butyllithium (initiator) in the cyclohexane solvent [11]. According to the different contents of polystyrene and polybutadiene, as well as the difference in the molecular structure, SBS can be divided into the linear and star structures, as shown in Figure 1 [12]. In general, the molecular weight of the star structure is higher than that of the linear structure. Previous research and applications have suggested that the SBSMA content in many countries is 3.0–6.0%, considering the limitations of asphalt pavement construction costs and construction technologies [13,14]. Yet, no in-depth investigation has been conducted on the higher dosage of this material. However, porous asphalt concrete has been more and more used in pavement materials with the advancements in pavement green technology [15,16]. Therefore, modified asphalt with high viscosity is commonly applied in porous asphalt concrete [17,18]. At present, modified asphalt with high viscosity is mainly fabricated using a high content of SBSMA (6.0–12.0%) [19].

Zhang et al. examined the rheological behavior of high-content SBSMA after the addition of a plasticizer (fural exact oil) and a crosslinker (sulfur). Their results illustrated that the plasticizer reduced the anti-rutting performance of SBSMA. The inclusion of a crosslinker created a polymer network, a network structure with better ageing, which increased SBSMA’s ageing resistance [20]. This is because the ageing of the polymer depends on the actual 3D structure (more chaotic or more structured) and the type of crosslinks [21]. However, the SBS modifier content in their study was just 6.0%, remarkably lower than what is used in practice. Yan et al. evaluated the ageing properties of high-content SBSMA, whose findings revealed that the SBS modifier breakdown occurs during the early stages of ageing. In addition, short-term ageing at elevated temperatures can severely degrade high-content SBSMA’s anti-rutting capabilities [22]. Lin et al. investigated the rheological properties of high-content SBSMA. Their results suggested that SBSMA with higher content would possess superior rheological qualities; however, 9% is the ideal dosage for economic reasons [23]. Zhang et al. explored the composition of high viscosity–modified asphalt and discovered that increasing the SBS modifier concentration is one of the most effective approaches to maintaining the physical qualities of high viscosity–modified asphalt [24]. Giacomo et al. observed that a high-content SBS modifier was able to lessen the ageing-induced stiffening of SBSMA. Compared to typical SBSMA with a lower polymer concentration, the SBS modifier network in the high-content SBSMA could present a barrier to the oxidation of the binder, leading to better anti-ageing performance [3,25].

**Figure 2:**
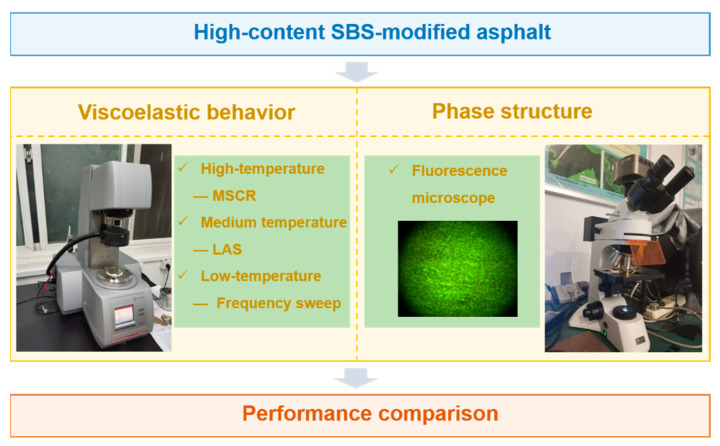
Flowchart of this study.

In a nutshell, limited studies on high-content SBSMA have been conducted so far. Furthermore, most research focuses on the influence of ageing on the properties of high-content SBSMA. At present, research on the viscoelastic properties and phase structure of high-content SBSMA is not detailed enough. Therefore, to investigate the viscoelastic behavior of high-content SBSMA at high to low temperatures, we employed three new dynamic shear rheometer (DSR) test methods. That is, the multiple stress creep recovery (MSCR) test, linear amplitude sweep (LAS) test, and 4 mm low-temperature frequency sweep test. Meanwhile, the microstructure of SBSMA with different contents was analyzed by a fluorescence microscope. The flowchart of this study is shown in Figure 2. This study clarified the viscoelastic properties and microscopic phase structure of high-content SBS modified asphalt at different temperatures, which provides a reference for the wider application of high-content SBS modified asphalt.

## 2. Materials and Methods

### 2.1. Raw Materials

Shell 70# neat asphalt was utilized to prepare high-content SBSMA. Its basic attributes are tabulated in Table 1. The adopted SBS modifiers were provided by Baling Petrochemical Company of Sinopec Group. Table 2 lists the basic parameters of the modifier. The modifier content was chosen as 3.0%, 4.5%, 6.0%, 7.5%, 9.0%, 10.5% and 12.0% of the mass of the neat asphalt, respectively. The sulfur powder with purity of more than 99% provided by Shanghai Qunkang Asphalt Technology Co., Ltd. (Shanghai, China) was selected as the stabilizer. The content of the stabilizer was chosen as 0.15% of the mass of the neat asphalt.

### 2.2. Experimental Methods

#### 2.2.1. Preparing SBSMA

**Figure 3:**
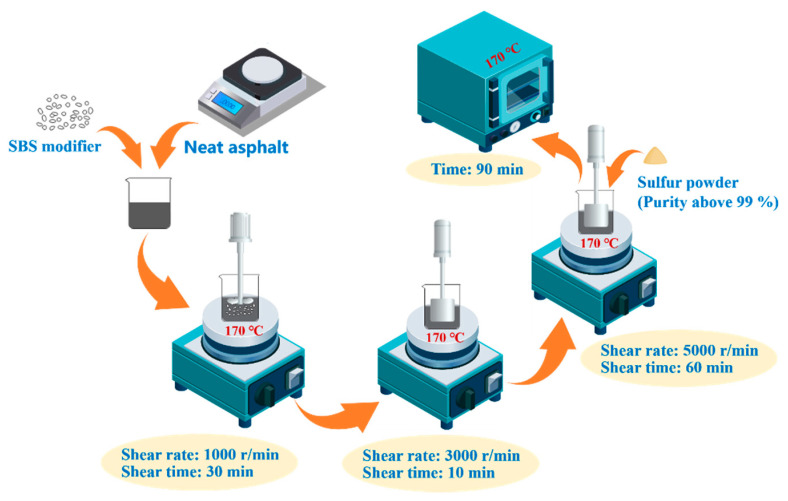
Preparation process of SBSMA.

SBSMA can be prepared as Figure 3. First, the corresponding quality of neat asphalt, SBS modifier, and stabilizer was weighed. We heated the neat asphalt to the molten state in an oven. The weighed SBS modifier was added into it and stirred with a vane stirrer at 170 °C and 1000 r/min for half an hour. The blended neat asphalt and SBS modifier were then sheared by a shear emulsifying machine at 170 °C and 3000 rotations per min for 10 min. Next, the stabilizer was added, and the mixture was sheared for an hour at 170 °C and 5000 rotations per min. Finally, the sheared SBS modifier was heated at 170 °C for 90 min to allow the prepared SBSMA to fully develop and escape the air bubbles created during the preparation process.

#### 2.2.2. MSCR Test

The high-temperature viscoelastic behavior of SBSMA was investigated using MSCR tests. In this test, Smartpave 102 DSR (Anton Paar company, Graz, Austria) was utilized. Two round parallel plates with a diameter of 25 mm were utilized, and the gap between them was 1 mm. To simulate the high temperature experienced by the asphalt pavement in summer, referring to the AASHTO M332-19 [26] classification standard, 64 °C was selected for the MSCR test. The creep and recovery tests were performed with the constant stress (0.1 kPa and 3.2 kPa), respectively. During the test, the stress was first loaded for 1 s. Afterward, zero stress was recovered for 9 s. First, the test was run for 20 cycles at 0.1 kPa. Then, it ran for 10 cycles at 3.2 kPa. Finally, it ran 30 creep and recovery cycles for 300 s [27,28].

#### 2.2.3. LAS Test

The LAS test can assess SBSMA’s fatigue properties at medium temperatures. Smartpave 102 DSR (Anton Paar company, Graz, Austria) was used in this test, with an 8 mm-parallel plate die and a 2 mm-gap. The LAS test was performed in a loading mode (controlled strain) with a design test time of 300 s. During the test, the sine wave dynamic load amplitude rose linearly from 0.1% to 30% [29,30]. Herein, 25 °C was chosen as the LAS test temperature.

#### 2.2.4. Low-Temperature Frequency Sweep Test

The low-temperature frequency sweep test was performed by DSR, which could overcome the disadvantages of too many materials and the long test time of the low-temperature bending beam rheometer (BBR) test. The 4 mm parallel plate low-temperature frequency sweep test can substitute the BBR test to assess the asphalt property at low temperatures [31]. Therefore, a 4 mm parallel plate for the low-temperature frequency sweep test was selected herein to explore SBSMA’s low-temperature viscoelastic behavior. The tests used a SmartPave Model 102 DSR with a 4 mm-parallel plate die placed at the 3 mm-gap. The loading model was controlled strain, and the strain was 1%; the sweep frequency was 0.1–100 rad/s, and the test temperature was −5 °C and −15 °C [32].

#### 2.2.5. Fluorescence Microscope Test

An LW300LFT fluorescence microscope (Nikon Corporation, Tokyo, Japan) was employed to determine SBSMA’s distribution and morphological characteristics in the asphalt phase. The magnification of the eyepiece of the fluorescence microscope and that of the objective lens was 10.

## 3. Results and Discussion

### 3.1. Viscoelastic Behavior at High Temperatures

#### 3.1.1. Non-Recoverable Creep Compliance and Creep Recovery Rate

Generally, a high-temperature rutting phenomenon will occur on the asphalt pavement due to the accumulation of the asphalt binder’s non-recoverable strain. Therefore, the non-recoverable creep compliance (*J_nr_*) is a critical evaluation indicator of the MSCR test (Equation (1)). Under a certain recovery time, the greater the recovery deformation and the smaller the amount of non-deformation of asphalt, the less likely high-temperature rutting will occur. Therefore, the creep recovery rate (*R*) can also represent the high-temperature viscoelastic behavior of asphalt (Equation (2)). *J_nr_* and *R* are calculated by average values in 10 creep recovery cycles, respectively [31]. The *J_nr_* at 0.1 kPa and 3.2 kPa are denoted as *J_nr_*0.1 and *J_nr_*3.2. Furthermore, the *R* at corresponding stress levels are represented as *R*0.1 and *R*3.2.

(1) $$J_{nr}=\frac{\varepsilon_{u}}{\sigma }$$

where, *ε_u_* is the adjusted strain value after the recovery period, *σ* is the value of the applied stress level.

(2) $$R=\frac{\varepsilon_{p}-\varepsilon_{u}}{\varepsilon_{p}}\;\times \;100\%$$

where, *ε_p_* is the adjusted strain value when the creep loading ends, *ε_u_* is the adjusted strain value after recovery period.

**Figure 4:**
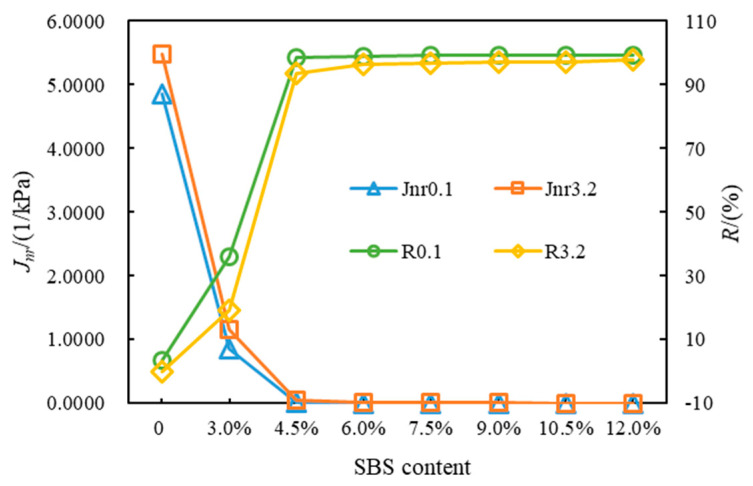
Non-recoverable creep compliance and creep recovery rate of asphalt.

**Figure 5:**
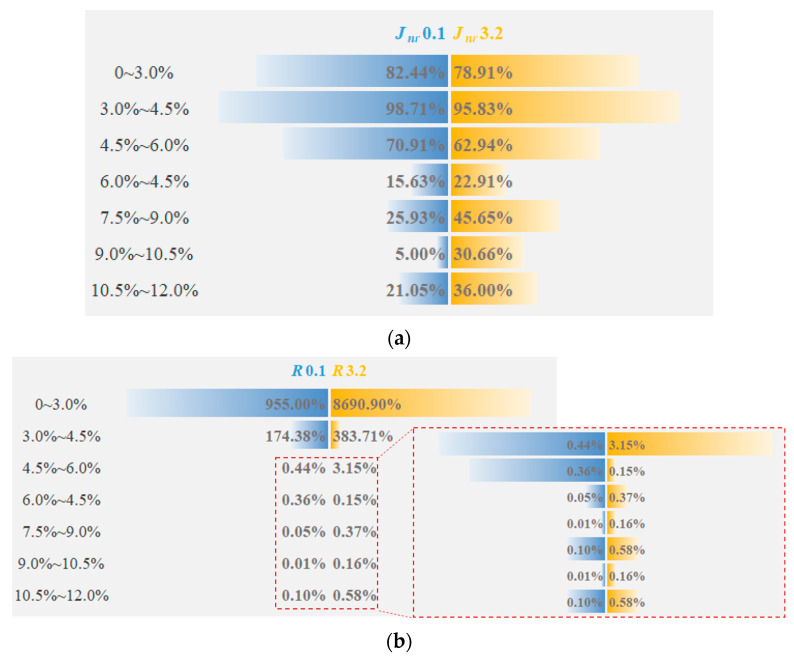
The change rate of *J_nr_* and *R*. (**a**) non-recoverable creep compliance; (**b**) Creep recovery rate.

Figure 4 presents the *J_nr_* and *R* of the eight kinds of asphalt at 64 °C. Under different stress levels, *J_nr_*3.2 > *J_nr_*0.1, *R*3.2 < *R*0.1. This order indicates that the increase in stress will worsen the high-temperature viscoelastic properties of SBSMA. With the rising modifier content, the *J_nr_* of SBSMA dropped and the *R* increased, suggesting that increasing the modifier content can improve the rheological properties of SBSMA. When the modifier content increased by 1.5%, an obvious turning point was noticed in the change in *J_nr_* and *R*, which was bounded by 4.5% content. The changing slope of the content less than 4.5% is much higher than that of the content greater than 4.5%, indicating that when the modifier content exceeds 4.5%, it can enhance the SBSMA’s high-temperature viscoelastic behavior with a limited improvement effect. This is because when the content of SBS modifier is 4.5%, the viscosity of the asphalt phase and the elasticity of the SBS phase in SBSMA reach an equilibrium state. Although increasing the content of the modifier can increase the mechanical strength of the SBS phase, the effect is not obvious. Figure 5 shows that for Shell 70# neat asphalt, the addition of the star SBS modifier to 3.0% content caused a decrease in *J_nr_*0.1 and *J_nr_*3.2 by 82.44% and 78.91%. *R*0.1 and *R*3.2 rose by 955.00% and 8690.90%. It is shown that adding the SBS modifier can considerably boost the neat asphalt’s high-temperature viscoelastic performance. For SBSMA, when it rises from 3.0% to 4.5%, the change rate of *J_nr_* and *R* is the largest, followed by the change rate when the content increases from 4.5% to 6.0%. Thereafter, with the increasing content, the change rate of *J_nr_* and *R* becomes smaller, which may be related to the microstructure of SBSMA.

#### 3.1.2. Stress Sensitivity

**Figure 6:**
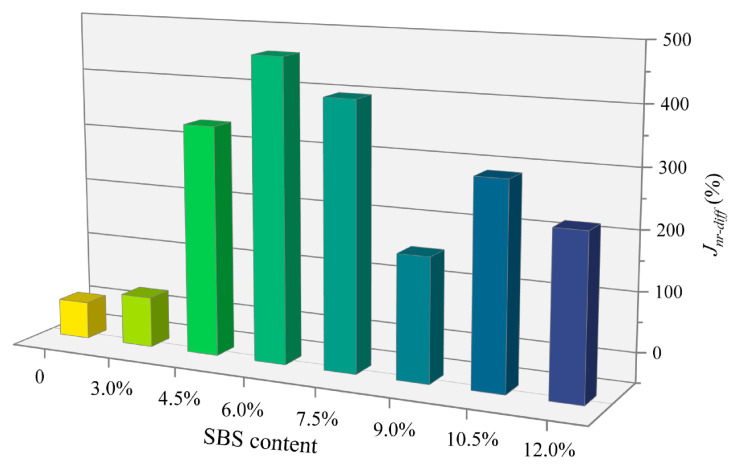
*J_nr-diff_* of eight types of asphalt.

The SBSMA’s sensitivity to stress can be expressed by the difference in the *J_nr_* under 3.2 kPa and 0.1 kPa, which is calculated according to Equation (3).

(3) $$J_{nr\text{-}diff}=\left[\left(J_{nr}3.2-J_{nr}0.1\right){/J}_{nr}0.1\right]\;\times \;100\%$$

where, *J_nr-diff_* is the stress sensitivity, *J_nr_*0.1 and *J_nr_*3.2 are the values of *J_nr_* of asphalt at 0.1 kPa and 3.2 kPa. The *J_nr-diff_* of eight types of asphalt at 64 °C are plotted in Figure 6.

Figure 6 shows that the *J_nr-diff_* of the neat asphalt is the smallest among the eight types of asphalt, and the *J_nr-diff_* of SBSMA is much larger than that of the neat asphalt. For SBSMA, *J_nr-diff_* has no evident change rule with the rising star SBS modifier content. The *J_nr-diff_* of 3.0% content is the smallest, and the *J_nr-diff_* of 6.0% content is the largest. This may be attributed to a large number of polymer chain segments inside the SBSMA with complex mechanical behavior and different phase structures. The literature has shown that the viscoelastic performance of modified asphalt could be determined according to the notion of whether the *J_nr-diff_* of the modified asphalt is greater than 5% [30]. When *J_nr-diff_* is greater than 5%, it is a nonlinear viscoelastic state. Similarly, when *J_nr-diff_* is less than 5%, it is a linear viscoelastic state. At 3.2 kPa and 64 °C, the viscoelastic performance of star SBSMA with a content of more than 3.0% is all nonlinear.

### 3.2. Viscoelastic Properties at Medium Temperatures

#### 3.2.1. Stress–Strain Response

**Figure 7:**
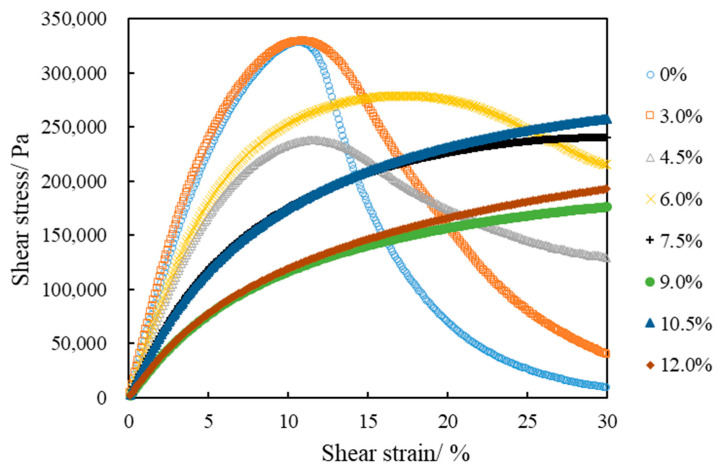
Stress–strain response of asphalt.

Asphalt undergoes elastic and plastic deformations in the LAS test under repeated loading. The shear stress gradually decreases when the applied load reaches a particular point; however, the shear strain increases in that case. A peak value of the shear stress is observed in the LAS test’s stress–strain curve. The AASHTO TP 101-12 specification defines this peak value as the asphalt yield stress, and its shear strain the yield strain. Figure 7 presents the stress–strain curves of LAS tests of eight kinds of asphalt. The neat asphalt has the highest yield stress and the smallest yield strain. Only the SBSMA with 3.0%, 4.5% and 6.0% contents demonstrated a peak in the stress–strain curve for the SBSMA. The order of yield stress was 3.0% > 6.0% > 4.5%, and the order of yield strain was 6.0% > 4.5% > 3.0%. The ranking of yield stress and yield strain is inconsistent. When the content was greater than 6.0%, the stress increases relatively slowly in the loading process. With the increase in strain, the stress gradually becomes flat and the stress–strain curve does not show a peak. This finding indicated that the yield stress or yield strain can only characterize the stress–strain response of asphalt under medium temperature conditions and repeated loadings but cannot characterize the fatigue properties of asphalt.

#### 3.2.2. Fatigue Life

The results of the LAS tests were further analyzed using the Viscoelastic Continuous Damage Mechanics theoretical model [31]. Notably, the LAS test is composed of frequency sweep and amplitude sweep. First, the parameter α is acquired via the frequency sweep test and is calculated according to Equations (4)–(6) [32,33].

(4) $$G^{\prime }=\left|G^{*}\right|\;\times \;\cos\delta$$

(5) $$\mathrm{lg}G^{\prime }=m(\mathrm{lg}\omega )+b$$

(6) $$a=\frac{1}{m}$$

where $G^{\prime }$ is the storage modulus (MPa), $G^{*}$ is the complex shear modulus (MPa), δ is the phase angle (°), *m* and *b* are the fitting parameters, *a* is a parameter for asphalt’s viscoelastic behavior.

Secondly, the damage variable (*D*) is calculated, as shown in Equation (7) [34].

(7) $$D\;(t)\;≅\overset{N}{\underset{i=1}{\sum }}{{[\pi \gamma }_{0}^{2}{\;(C}_{i-1}-C_{i})]}^{a/(1+a)}{{(t}_{i}-t_{i-1})}^{1/(1+a)}$$

where *t* is the test time (s), *t_i_* is the current test time, *t_i_*_−1_ is the previous test time, *N* is the total number of tests, *C*(*t*) is the integrity parameters, which is calculated by Equation (8).

(8) $$C\;(t)=\left|G^{*}\right|(t)/{\left|G^{*}\right|}_{\mathrm{initial}}$$

where, $\left|G^{*}\right|\left(t\right)$ is the complex shear modulus with test time in the amplitude sweep test (MPa), ${\left|G^{*}\right|}_{\mathrm{initial}}$ is the complex shear modulus when the test starts (MPa), $\gamma_{0}$ is the test strain (%).

For the loss variable (*D* (*t*)) and integrity parameter (*C* (*t*)), there is the following relationship, as shown in Equation (9).

(9) $${C\;(t)=C}_{0}-C_{1}{\left[D\;(t)\right]}^{C_{2}}$$

where, $C_{0}=1$, $C_{1}$ and $C_{2}$ are the fitting parameters.

Finally, the fatigue life is calculated, as shown in Equation (10).

(10) $$N_{f}=A\;(\gamma_{\max}{)}^{-B}$$

where, $\gamma_{\max}$ is the expected maximum strain (%) and *A* and *B* are the fatigue correlation coefficients, which are calculated according to Equation (11) to Equation (12).

(11) $$A=\frac{f\;{\left(D_{f}\right)}^{[1+(1-C_{2})a]}}{[1+(1-C_{2}{)a](\pi C}_{1}C_{2}{)}^{a}}$$

where, *f* = 10 Hz, $D_{f}=(\frac{C_{0}-C_{\mathrm{peak}}}{C_{1}}{)}^{{1/C}_{2}}$.

(12) B=2a

**Figure 8:**
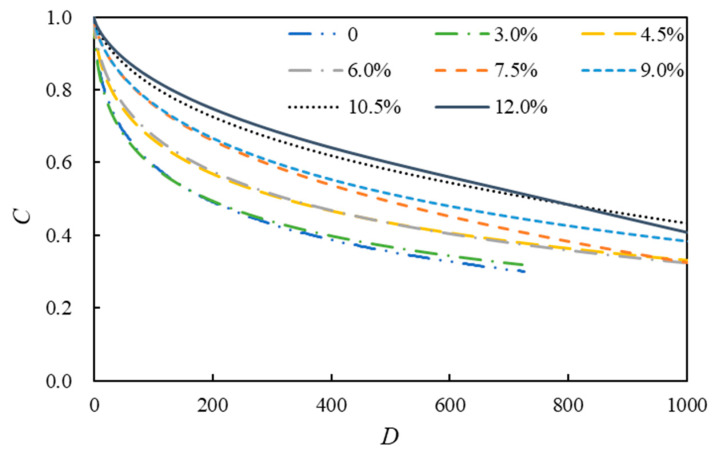
Damage characteristic curves of asphalt.

**Figure 9:**
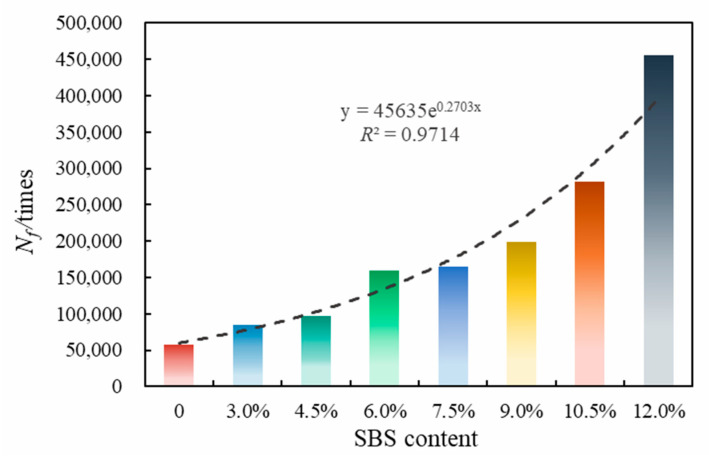
Fatigue life of asphalt.

The above calculation results present the damage characteristic curves and fatigue life curves of eight kinds of asphalt (Figure 8 and Figure 9).

Figure 8 shows that *C* represents the integrity parameter of asphalt and *D* represents the cumulative damage parameter. The expression *C* = 1 denotes that the asphalt is intact, and the expression *C* = 0 denotes that it has been completely damaged. Figure 8 shows that when the cumulative damage parameter has a certain value, the asphalt’s integrity improves with the rising modifier content. Among them, the neat asphalt’s integrity is the worst, and the integrity of the 12.0% SBSMA is the best. However, the damage characteristic curves of 12.0% and 10.5% SBSMA appear staggered, suggesting that with the increase in the damage intensity, the damage resistance of 10.5% SBSMA is higher than that of 12.0%.

Figure 9 shows different asphalts’ fatigue life at 2.5% strain. The fatigue life of SBSMA increases exponentially with the increasing modifier content. This is because with the increase in SBS modifier content, the distribution of the SBS phase in asphalt gradually forms a cross-linking state, which increases the fatigue life. Specifically, for Shell 70# neat asphalt, *N_f_* increased by 47.3% after adding 3.0% of the star SBS modifier. For SBSMA, when the content rises from 4.5% to 6.0%, the change rate of *N_f_* is the largest (63.9%), followed by the change rate from 10.5% to 12.0%, and the change rate from 6.0% to 7.5% is the smallest (2.8%).

### 3.3. Viscoelastic Properties at Low Temperatures

#### 3.3.1. Modulus and Phase Angle

**Figure 10:**
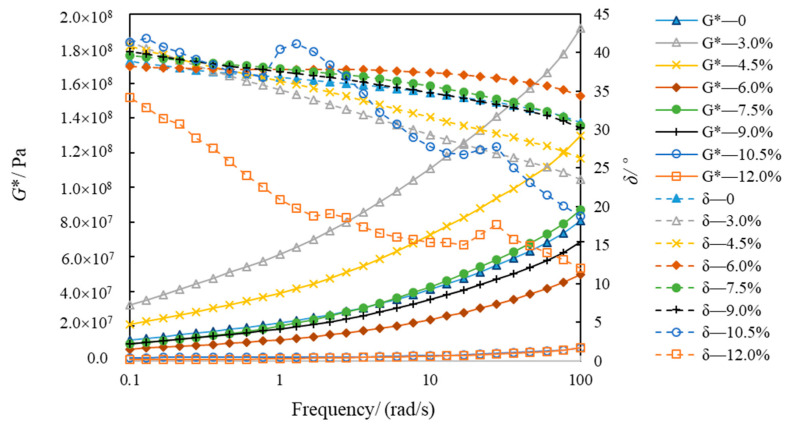
Modulus and phase angle of asphalt at −5 °C.

**Figure 11:**
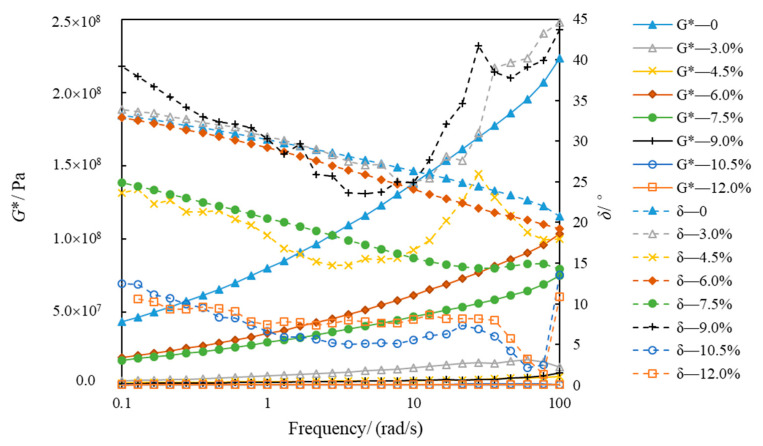
Modulus and phase angle of asphalt at −15 °C.

Figure 10 and Figure 11 show the variations in G* and *δ*, with eight kinds of asphalt frequencies at −5 °C and −15 °C. The G* of eight types of asphalt increased when the frequency was gradually augmented from 0.1 rad/s to 100 rad/s, which conformed to Generalized Maxwell model [35]. However, the phase angle change is more complicated. At −5 °C, the phase angle of SBSMA decreases with the increasing frequency when the content of SBSMA is less than 10.5%. Although the SBSMA with contents of 10.5% and 12.0%, the phase angle decreased and the phenomenon of “first increase and then decrease” will appear in the change process. At −15 °C, the phase angle of SBSMA has no obvious pattern when the frequency enlarges. With the rising modifier content, the G* and *δ* of SBSMA had little change. The order of the G* and *δ* of the eight types of asphalt at −5 °C and −15 °C was inconsistent. This may be because in the preparation of high-content SBSMA, the same shear time as the low-content SBSMA was used, resulting in a portion of the SBS of the high-content SBSMA being too late to swell and cross-link. Additionally, each high-content SBSMA is too late to swell and the cross-link of the SBS is different, so the SBSMA with the increase in the amount of low-temperature viscoelastic properties is also different.

#### 3.3.2. Evaluating Index

Sui et al. [36] compared the relaxation modulus *G* (*t*) master curve according to the 4 mm DSR test with the creep stiffness modulus *S* (*t*) master curve based on BBR tests. They showed that *G* (60 s) and *m_r_* (60 s) had an excellent linear correlation with *S* (60 s) and *m_c_* (60 s). Therefore, *G* (60 s) and *m_r_* (60 s) are recommended for 4 mm DSR as the evaluation indicator of low-temperature viscoelastic properties of asphalt. While calculating *G* (60 s) and *m_r_* (60 s), the primary curve of storage modulus (*G*′) of asphalt was first derived by fitting the time-temperature equivalence principle. Equation (13) describes the transformation between the primary curves of the $G^{\prime }\;(\omega )$ and the *G* (*t*). Then, Equation (14) was adopted to fit the master curve of the *G* (*t*). Finally, *G* (60 s) and *m_r_* (60 s) were counted according to Equations (15) and (16), respectively.

(13) $$G\;(t)\;\approx \;{G^{\prime }\;(\omega )\;|}_{\omega =2/\pi t}$$

where, G (t) is the relaxation modulus (Pa), $G^{\prime }\;(\omega )$ is the storage modulus (Pa), *t* is the test time (s), *ω* is the angular frequency (rad/s).

(14) $$y=ax^{2}+bx+c$$

where, *a*, *b*, and *c* are parameters for fitting.

(15) $${G\;(60\;s)=ax}^{2}+bx+{\;c\;|}_{x=1.78}$$

(16) $$m_{r}\;(60\;s)=2ax+{\;b\;|}_{x=1.78}$$

**Figure 12:**
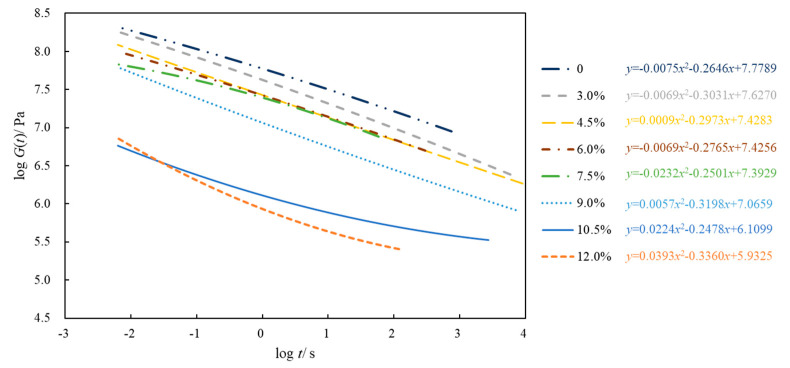
Main curve of relaxation modulus of asphalt at low temperatures (−15 °C).

**Figure 13:**
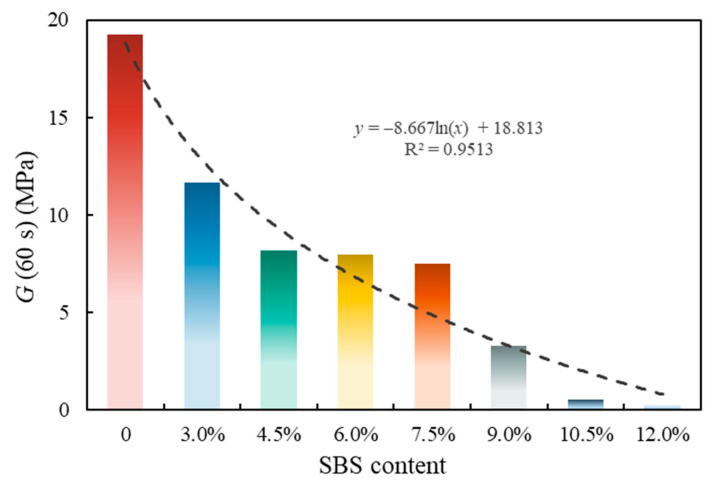
Evaluation index *G* (60 s) of asphalt at low temperatures.

**Figure 14:**
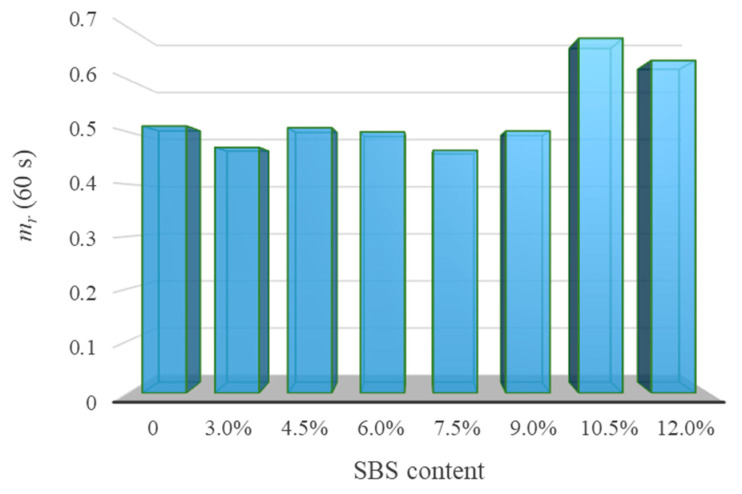
Evaluation index *m_r_* (60 s) of asphalt at low temperatures.

Using −15 °C as the reference temperature, the master curve of the relaxation modulus of eight kinds of asphalt is drawn in this study (Figure 12). Next, *G* (60 s) and *m_r_* (60 s) were counted (Figure 13 and Figure 14).

Figure 12 plots that the relaxation modulus level of neat asphalt is obviously higher than that of SBSMA, whereas the relaxation modulus of 3.0% is the largest and that of 12.0% is the smallest. Furthermore, the SBS modifier reduced the asphalt’s relaxation modulus level, lowering the temperature stress accumulation inside the asphalt and improving its cracking resistance at low temperatures.

Figure 13 shows the low-temperature property evaluation index *G* (60 s) of asphalt. It is shown that the *G* (60 s) of SBSMA decreased logarithmically with the increasing modifier content. For Shell 70# neat asphalt, *G* (60 s) decreased by 39.5% after adding 3.0% content. For SBSMA, when it rises from 9.0% to 10.5%, the change rate of *G* (60 s) is the largest. In contrast, the change rate is the smallest when the content increases from 4.5% to 6.0%. Figure 14 shows the asphalt’s low-temperature property evaluation index *m_r_* (60 s). With the increasing SBS modifier content, *m_r_* (60 s) shows no apparent change law, which is attributed to the different slopes of relaxation modulus curves of different types of asphalt in 60 s. Importantly, using *m_r_* (60 s) to determine the low-temperature property of SBSMA is still worthy of in-depth discussion and research.

### 3.4. Phase Structure of SBSMA

**Figure 15:**
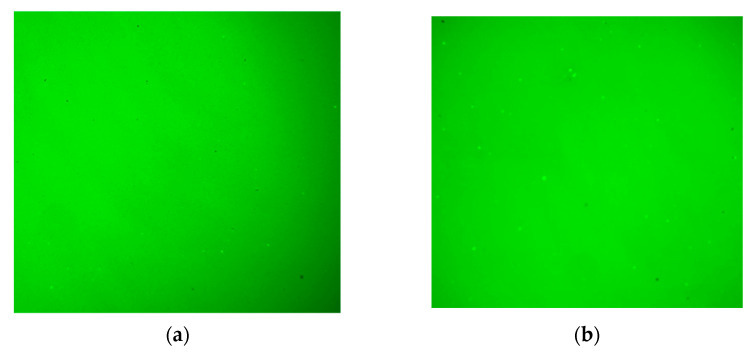

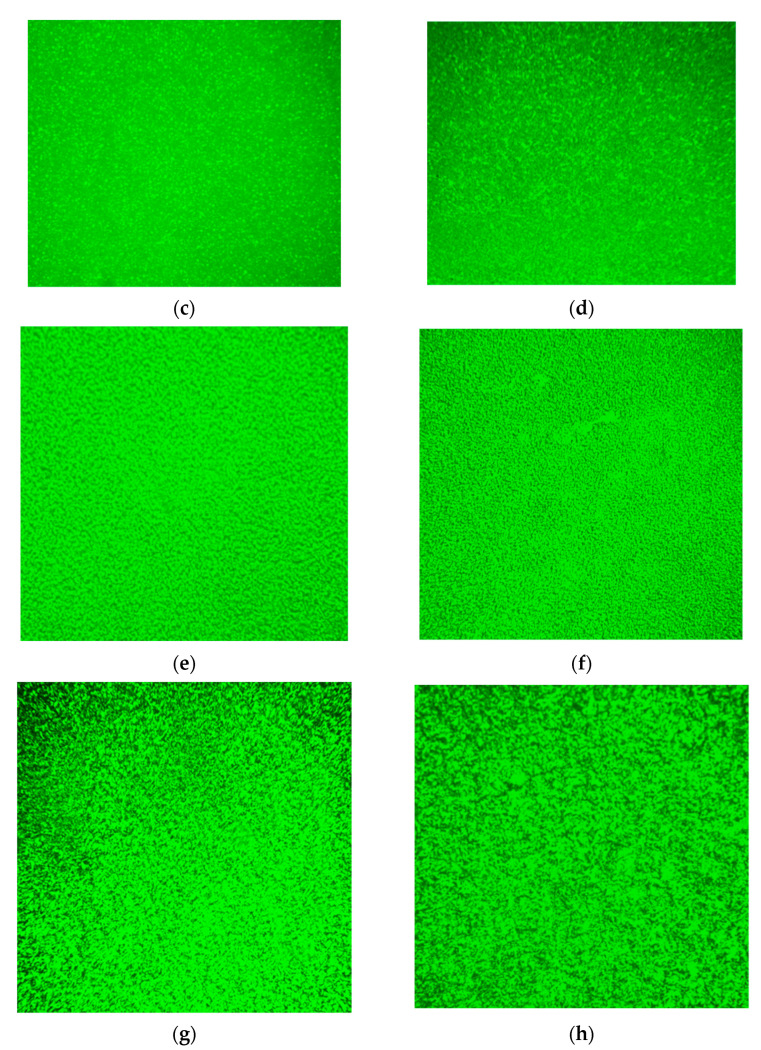
Fluorescent microscopic images of asphalt. (**a**) Neat asphalt, (**b**) 3.0% SBSMA, (**c**) 4.5% SBSMA, (**d**) 6.0% SBSMA, (**e**) 7.5% SBSMA, (**f**) 9.0% SBSMA, (**g**) 10.5% SBSMA, (**h**) 12.0% SBSMA.

Figure 15 illustrates the microscopic phase structures of the eight kinds of asphalt under a 100-fold fluorescence microscope.

The microscopic phase structure of SBSMA is a two-phase (SBS phase and asphalt phase) coexistence of the co-blended structural system compared to the neat asphalt. The phase structure varies with the SBS modifier content. When the SBS modifier content rises from 3.0% to 12.0%, the distribution state of SBS phase in asphalt phase gradually forms a cross-linking state; that is, the SBS modifier gradually changes from the dispersed to the continuous phase state. This is because as the modifier increases, the number of particles of the modifier increases, and its specific surface area increases significantly. Under the action of surface tension, the modifier particles are more likely to agglomerate. Specifically, when this content is 3.0% and 4.5%, the SBS modifier is in the dispersed phase state, whereas the neat asphalt is in the continuous phase state. The 4.5% content of the SBS modifier dispersion is more uniform than the 3.0% content of the SBS modifier. When it reaches 6.0%, the SBS modifier is also present in the dispersed phase. Moreover, a particular network structure appears and the SBS modifier gradually changes from the dispersed to the continuous phase state. When the SBS modifier content is 7.5% and 9.0%, the two phases of the blend are continuous, and the modifier-formed network structure is intertwined with each other. Compared with the 7.5% content of the SBS modifier, the mesh structure formed by the 9.0% content is more closely intertwined. When this content reaches 10.5% and 12.0%, the SBS modifier becomes a continuous phase state, whereas the neat asphalt becomes a dispersed phase state, and the formed network structure exhibits a rough surface and large interwoven “leaf”. In contrast, the 12.0% SBSMA had a rougher surface and larger interwoven “blade” than the 10.5% SBSMA.

## 4. Conclusions

(1) With the increasing SBS modifier content (3.0–12.0%), the non-recoverable creep compliance of SBSMA drops with the growing creep recovery rate. The modifier content increases the high-temperature viscoelastic performance of SBSMA. An obvious turning point was observed in the change in non-recoverable creep compliance and creep recovery rate, which is bounded by the 4.5% content. The changing slope of the content less than 4.5% is much higher than that of the content greater than 4.5%.
(2) The fatigue life of SBSMA increases exponentially with the increasing modifier content. Moreover, the growth rate of fatigue life is the largest (63.9%) when the content increases from 4.5% to 6.0%.
(3) The 4 mm DSR test can evaluate the viscoelastic performance of SBSMA at low temperatures. *G* (60 s) and *m_r_* (60 s) were selected as evaluation indicators. The *G* (60 s) of SBSMA decreases logarithmically with the increasing modifier content (3.0–12.0%). However, *m_r_* (60 s) has no noticeable change with the rising content. The SBS modifier cut down the low-temperature relaxation modulus of asphalt.
(4) In terms of the microscopic phase structure, the microscopic phase structure of SBSMA is a two-phase (SBS modifier and neat asphalt) coexistence of the co-blended structural system. With the increasing SBS modifier content, the SBS modifier gradually changes from a dispersed to a continuous phase state. When the modifier amount is less than 6.0%, the SBS modifier is present in a dispersed phase. Similarly, when the modifier is more than 6.0%, the SBS modifier is present in the continuous phase.
(5) The viscoelastic properties of high-content SBS modified asphalt at different temperature were investigated, and the phase structure of different content SBS modified asphalt were clarified. The chemical composition of high-content SBS modified asphalt and its aging characteristics will be studied in the next step.

## Figures and Tables

**Table 1:** Basic property of neat asphalt.

Item	Shell 70#
Penetration at 25 °C, 0.1 mm	70.8
Softening point, °C	49.2
Ductility at 5 °C, 5 cm min^−1^, cm	74.3

**Table 2:** Technical performance of SBS modifier.

Type	Star Type
Specific gravity, g cm^−3^	0.94
Elongation at break, %	680
Tensile strength, MPa	21.2
Melt index, g (10 min^−1^)	7.0

## Data Availability

Access to any other materials can be requested by writing to the corresponding authors.
